# Hereditary Hemorrhagic telangiectasia: a rare familial case with delayed diagnosis despite decades of recurrent bleeding

**DOI:** 10.1093/omcr/omaf189

**Published:** 2025-09-28

**Authors:** Ashutosh Vardhan Rahi, Abhishek Kumar, Jitendra Singh, Nilesh Kumar, Kailash Kumar, Anju Dinkar

**Affiliations:** Department of General Medicine, Institute of Medical Sciences, Banaras Hindu University, Varanasi, Uttar Pradesh, 221005, India; Department of General Medicine, Institute of Medical Sciences, Banaras Hindu University, Varanasi, Uttar Pradesh, 221005, India; Department of General Medicine, Institute of Medical Sciences, Banaras Hindu University, Varanasi, Uttar Pradesh, 221005, India; Department of Medicine, King George's Medical University, Lucknow, Uttar Pradesh, 226003, India; Department of General Medicine, Institute of Medical Sciences, Banaras Hindu University, Varanasi, Uttar Pradesh, 221005, India; Department of General Medicine, Institute of Medical Sciences, Banaras Hindu University, Varanasi, Uttar Pradesh, 221005, India; Department of Microbiology, Sanjay Gandhi Postgraduate Institute of Medical Sciences, Lucknow, Uttar Pradesh, 226014, India

**Keywords:** ACVRL1 mutation, Osler-weber-Rendu syndrome (OWRS), Curaçao criteria, recurrent epistaxis, antiangiogenic therapy

## Abstract

Hereditary Hemorrhagic Telangiectasia (HHT), or Osler-Weber-Rendu syndrome, is a rare autosomal dominant vascular disorder characterized by mucocutaneous telangiectasias and visceral arteriovenous malformations (AVMs). We report the case of a 45-year-old male with a 20-year history of recurrent spontaneous epistaxis, chronic fatigue, and iron deficiency anemia, unresponsive to repeated nasal cauterizations. A strong family history of similar bleeding episodes in his mother and son heightened clinical suspicion. Examination revealed severe pallor without visible telangiectasias. Upper gastrointestinal endoscopy demonstrated telangiectatic lesions, while imaging excluded pulmonary and cerebral AVMs. Genetic testing confirmed HHT type 2 by detecting a pathogenic *ACVRL1* gene variant. The patient was managed with intravenous iron, blood transfusions, topical nasal care, and laser therapy for refractory epistaxis. This case underscores the diagnostic challenges of HHT without classical telangiectasias and highlights the importance of early genetic testing and family screening to prevent serious complications.

## Introduction

Hereditary hemorrhagic telangiectasia (Osler-Weber-Rendu syndrome) is a rare autosomal dominant vascular disorder characterized by dysplastic, fragile vessels (vascular dysplasia) throughout multiple organ systems, which predisposes patients to recurrent bleeding. The hallmark vascular abnormalities typically present as telangiectasias on mucosal and cutaneous surfaces, along with AVMs involving visceral organs [1]. These lesions may involve the nasopharynx, central nervous system, lungs, liver, spleen, gastrointestinal (GI) and genitourinary tracts, and the conjunctiva, trunk, and extremities [2, 3]. These malformed vessels underlie the majority of bleeding complications in HHT, which may range from mild recurrent epistaxis to life-threatening intracranial hemorrhage [4–6]. Recurrent epistaxis, often beginning in the second decade of life, is the earliest and most common clinical feature of the disease. Additional complications may include pulmonary hypertension, thrombotic tendencies, and immune dysregulation in some individuals [7]. Although many patients present early, symptom onset may be delayed, with approximately 90% manifesting clinical characteristics by the age of year 40 [8, 9]. The disorder's burden progresses with age as the number and severity of telangiectasias increase, leading to a higher frequency of epistaxis or GI bleeding and subsequent iron-deficiency anemia.

The underlying pathophysiology of HHT involves mutations in genes such as ENG, ACVRL1, and SMAD4, which encode receptors within the transforming growth factor-beta (TGF-β) superfamily. These mutations impair downstream signaling, disrupting angiogenesis and endothelial cell function. As a result, the impacted arteries demonstrate compromised structural integrity, persistently dilated conditions, and increased susceptibility to rupture due to disrupted cytoskeletal organization and impaired vascular remodeling [10].

## Case report

A 45-year-old male, previously healthy and employed in a dye manufacturing unit, presented with a 25-year history of recurrent spontaneous epistaxis in the absence of any known chronic medical conditions. The bleeding episodes were not associated with local trauma, seasonal variation, or bleeding from other anatomical sites. He also reported generalized weakness and easy fatigability of similar duration. Notably, a positive family history was elicited, with his mother and son experiencing comparable symptoms. Despite multiple nasal cauterizations and the use of antifibrinolytic agents, his symptoms persisted without significant improvement.

The patient was severely pale on physical examination, but no telangiectatic lesions or ecchymoses were identified on the skin or mucosal surfaces. Systemic examination was otherwise unremarkable. The otolaryngological evaluation, which included anterior rhinoscopy, indicated no active nasal cause of haemorrhage.

**Figure 1:**
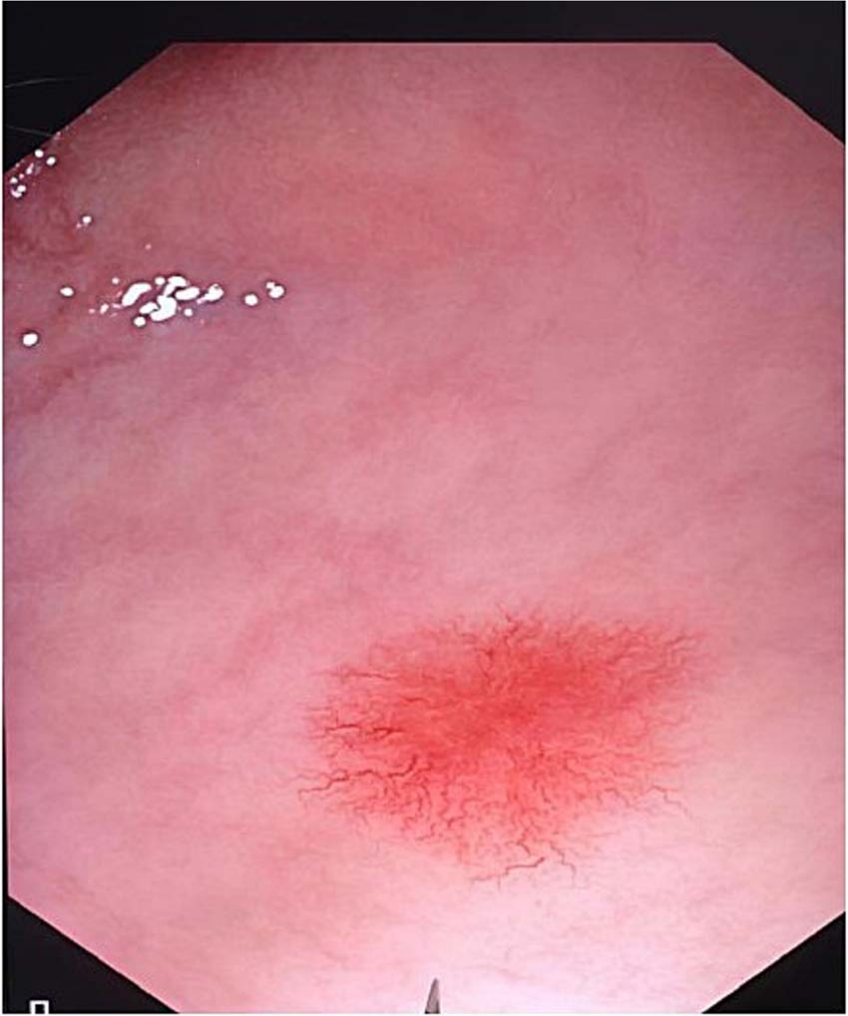
Upper GI endoscopy showing telangiectatic lesion.

Laboratory investigations demonstrated marked microcytic hypochromic anemia (hemoglobin: 6.2 g/dl, MCV: 67 fl), with low serum ferritin and elevated total iron-binding capacity, consistent with iron deficiency anemia. Coagulation parameters (PT, INR, aPTT), bleeding time, clotting time, and von Willebrand factor assay were all within normal ranges. Upper and lower gastrointestinal endoscopy showed multiple telangiectatic lesions (Fig. 1). Simultaneously, contrast-enhanced computed tomography (CT) angiography of the brain, thorax, and abdomen showed no evidence of visceral AVMs.

**Figure 2:**
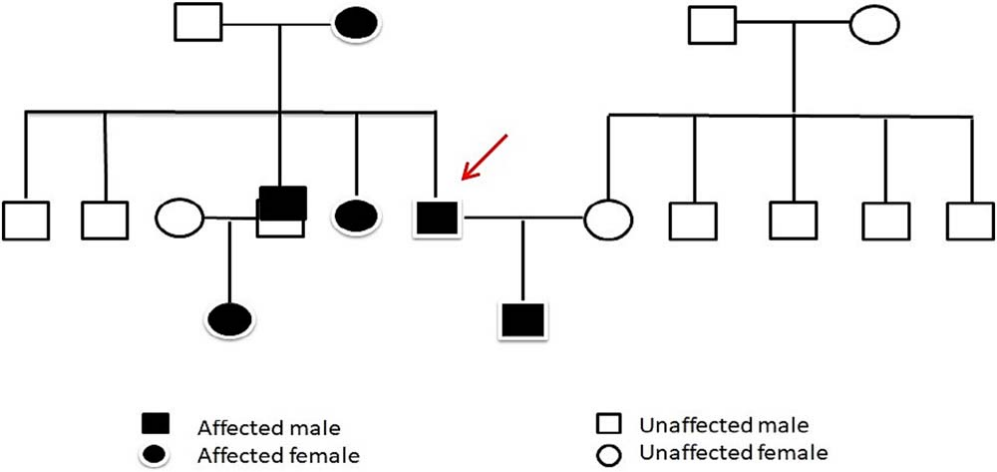
Pedigree chart of the present case.

Genetic testing was performed based on the patient's clinical background, which included a chronic history of spontaneous epistaxis, iron deficiency anemia, positive family history, and endoscopic findings. A disease-causing mutation in the *ACVRL1* gene (exon 9, chromosome 12) was detected, establishing the diagnosis of Hereditary Hemorrhagic Telangiectasia type 2 (HHT-2). A pedigree chart illustrated the autosomal dominant inheritance pattern (Fig. 2). The patient fulfilled more than three Curaçao criteria, thereby meeting the international consensus definition for a definitive diagnosis of HHT.

Management included intravenous iron therapy, numerous packed red blood cell transfusions, and oral iron supplements for ongoing iron deficiency anemia. Supportive treatment for epistaxis involved nasal humidification, saline sprays, and avoidance of mucosal dryness. Persistent telangiectasias were treated with endonasal laser therapy and electrocautery under otolaryngology supervision. Given the refractory bleeding and confirmed HHT2 mutation, systemic antiangiogenic treatment with bevacizumab was considered to mitigate vascular proliferation and bleeding frequency. Screening for visceral AVMs was completed, with plans for embolization of clinically significant lesions to prevent complications such as bleeding or high-output cardiac failure. A multidisciplinary care strategy involving hematology, otolaryngology, and interventional radiology was implemented to ensure holistic and longitudinal management.

## Discussion

HHT is a rare inherited vascular disorder marked by presence of mucocutaneous telangiectasias and arteriovenous malformations affecting internal organs. The clinical diagnosis of HHT is primarily guided by the Curaçao criteria, established in 1999 by the scientific advisory board of the HHT Foundation International to enhance patient care and ensure uniformity in clinical research.

The Curaçao diagnostic criteria consist of four key components: (1) spontaneous and recurrent epistaxis; (2) numerous mucocutaneous telangiectasias at characteristics sites such as nasal mucosa, lips, oral mucosa and fingertips; (3) the presence of visceral arteriovenous malformations (AVMs) involving organs such as gastrointestinal tract, lungs, liver, brain, or spinal cord; and (4) a positive family history in a first-degree relative who meets the same diagnostic criteria. The diagnostic certainty of HHT is stratified based on the number of Curaçao criteria met: a definite diagnosis requires three or more, a possible or suspected diagnosis requires two, and the diagnosis is considered unlikely if fewer than two criteria are fulfilled [6].

Based on the clinical presentation and examination, differential diagnosis includes clotting disorders such as von Willebrand disease and platelet function abnormalities, which were excluded by a normal coagulation profile and von Willebrand factor assay. Hematological conditions, including aplastic anemia and hematologic malignancies, were also considered but ruled out based on clinical findings and laboratory parameters [11]. Local structural nasal pathologies, chronic rhinosinusitis, and hereditary bleeding disorders were similarly evaluated and excluded.

No quantitative threshold is established for the frequency or severity of epistaxis necessary for diagnosis; however, the criteria highlight the significance of spontaneous and repeated episodes, especially those that occur nocturnally. With recurrent epistaxis, visceral gastrointestinal telangiectasias, and a positive first-degree family history, the patient met the threshold of three Curaçao criteria, establishing a confirmed diagnosis of HHT.

In this case, the patient’s son, who presented with similar clinical manifestations, was referred for genetic counseling followed by confirmatory genetic testing. He was found to carry the identical pathogenic mutation in the *ACVRL1* gene (exon 9, chromosome 12), thereby confirming the diagnosis of Hereditary Hemorrhagic Telangiectasia type 2 (HHT-2). Other first-degree relatives have similarly been advised to undergo detailed clinical assessment and appropriate genetic testing. This underscores the critical importance of cascade screening in families affected by HHT. The identification of at-risk relatives, including those who are asymptomatic, facilitates the early detection of visceral arteriovenous malformations (AVMs).

The management of Hereditary Hemorrhagic Telangiectasia (HHT) is mainly symptomatic and requires a multidisciplinary approach to control bleeding, correct anemia, and prevent complications from visceral arteriovenous malformations (AVMs).

1. Epistaxis and mucocutaneous telangiectasias: The most common symptom, recurrent epistaxis, is managed initially with conservative measures, including humidification, saline sprays, and topical lubricants, as well as topical oestrogen cream/ointment, antifibrinolytics, and iron supplementation for anemia. For persistent bleeding, local therapies such as cauterization, laser photocoagulation, septodermoplasty, and nasal artery embolisation and nasal cavity closure (Young’s procedure) may be considered [12].

2. Gastrointestinal bleeding: Endoscopic ablation techniques commonly treat gastrointestinal telangiectasias, including argon plasma coagulation and laser therapy. For refractory cases, systemic therapies such as hormonal therapy (estrogen-progesterone) have been used with variable success but are now less favored [12].

3. Visceral AVMs: Pulmonary AVMs require embolization to reduce the risk of stroke and brain abscess (4). Cerebral AVMs, depending on their size and location, may be treated with surgical resection, stereotactic radiosurgery, or embolotherapy. Hepatic AVMs are usually managed conservatively, with liver transplantation reserved for severe high-output cardiac failure or portal hypertension [12].

4. Systemic antiangiogenic therapy: Bevacizumab, a monoclonal antibody targeting vascular endothelial growth factor (VEGF), has emerged as an option in patients with severe, refractory bleeding or high-output cardiac failure secondary to hepatic AVMs. Clinical studies have demonstrated its efficacy in reducing transfusion requirements, improving hemoglobin levels, and decreasing cardiac output in selected patients [13].

Early identification of individuals with Hereditary Hemorrhagic Telangiectasia (HHT) is essential to prevent serious complications such as high-output cardiac failure or fatal hemorrhage from untreated arteriovenous malformations (AVMs). Family screening and genetic confirmation play a pivotal role, especially for at-risk relatives who may be asymptomatic but remain vulnerable to disease-related complications. When combined with clinical suspicion and a detailed family history, genetic testing enables timely diagnosis, targeted surveillance, and initiation of appropriate multidisciplinary care. HHT continues to present diagnostic and therapeutic challenges because of its diverse clinical features and potential for life-threatening outcomes. This case emphasizes the importance of considering HHT in patients with unexplained chronic epistaxis and demonstrates the value of early genetic testing. Novel targeted therapies, including antiangiogenic agents, show promise in controlling refractory bleeding and improving prognosis.
